# Balance Impairments as Differential Markers of Dementia Disease Subtype

**DOI:** 10.3389/fbioe.2021.639337

**Published:** 2021-03-11

**Authors:** Ríona Mc Ardle, Stephanie Pratt, Christopher Buckley, Silvia Del Din, Brook Galna, Alan Thomas, Lynn Rochester, Lisa Alcock

**Affiliations:** ^1^Translational and Clinical Research Institute, Newcastle University, Newcastle upon Tyne, United Kingdom; ^2^Population Health Sciences Institute, Newcastle University, Newcastle upon Tyne, United Kingdom; ^3^Department of Sport, Exercise and Rehabilitation, Northumbria University, Newcastle upon Tyne, United Kingdom; ^4^School of Biomedical, Nutritional and Sports Sciences, Newcastle University, Newcastle upon Tyne, United Kingdom; ^5^Newcastle upon Tyne Hospitals, National Health Service Foundation Trust, Newcastle upon Tyne, United Kingdom

**Keywords:** dementia, Alzheimer’s disease, Lewy body disease, Parkinson’s disease, balance, accelerometer, postural control

## Abstract

**Background:**

Accurately differentiating dementia subtypes, such as Alzheimer’s disease (AD) and Lewy body disease [including dementia with Lewy bodies (DLB) and Parkinson’s disease dementia (PDD)] is important to ensure appropriate management and treatment of the disease. Similarities in clinical presentation create difficulties for differential diagnosis. Simple supportive markers, such as balance assessments, may be useful to the diagnostic toolkit. This study aimed to identify differences in balance impairments between different dementia disease subtypes and normal aging using a single triaxial accelerometer.

**Methods:**

Ninety-seven participants were recruited, forming four groups: cognitive impairment due to Alzheimer’s disease (AD group; *n* = 31), dementia with Lewy bodies (DLB group; *n* = 26), Parkinson’s disease dementia (PDD group; *n* = 13), and normal aging controls (*n* = 27). Participants were asked to stand still for 2 minutes in a standardized position with their eyes open while wearing a single triaxial accelerometer on their lower back. Seven balance characteristics were derived, including jerk (combined, mediolateral, and anterior–posterior), root mean square (RMS; combined, mediolateral, and anterior–posterior), and ellipsis. Mann–Whitney *U* tests identified the balance differences between groups. Receiver operating characteristics and area under the curve (AUC) determined the overall accuracy of the selected balance characteristics.

**Results:**

The PDD group demonstrated higher RMS [combined (*p* = 0.001), mediolateral (*p* = 0.005), and anterior–posterior (*p* = 0.001)] and ellipsis scores (*p* < 0.002) than the AD group (AUC = 0.71–0.82). The PDD group also demonstrated significantly impaired balance across all characteristics (*p* ≤ 0.001) compared to the controls (AUC = 0.79–0.83). Balance differences were not significant between PDD and DLB (AUC = 0.69–0.74), DLB and AD (AUC = 0.50–0.65), DLB and controls (AUC = 0.62–0.68), or AD and controls (AUC = 0.55–0.67) following Bonferroni correction.

**Discussion:**

Although feasible and quick to conduct, key findings suggest that an accelerometer-based balance during quiet standing does not differentiate dementia disease subtypes accurately. Assessments that challenge balance more, such as gait or standing with eyes closed, may prove more effective to support differential diagnosis.

## Introduction

Assessing motor performance, such as gait and balance, in the aging population may be a useful clinical tool for predicting a range of clinical outcomes, such as falls risk, neurological disorders, cognitive impairment, and mortality (Fritz and Lusardi, 2009; Schoneburg et al., 2013; Creaby and Cole, 2018; Buckley et al., 2019; Modarresi et al., 2019; Peel et al., 2019). Recently, motor performance has been reported as a potential supportive marker of differentiating Lewy body disease (LBD) from Alzheimer’s disease (AD) (Fritz et al., 2016; Mc Ardle et al., 2019, 2020). Identifying supportive clinical tools to differentiate dementia subtypes, such as LBD [which includes dementia with Lewy bodies (DLB) and Parkinson’s disease dementia (PDD)], is of critical importance to ensure accurate and appropriate treatment and care provision for people with dementia (Palmqvist et al., 2009). This is particularly apparent for people with DLB, as DLB is underdiagnosed and may be misdiagnosed as AD due to similarities in clinical presentation (Palmqvist et al., 2009; Kane et al., 2018). As such, quick and easy-to-use diagnostic tools may be welcome additions to the clinician’s toolkit.

Motor assessments that require minimal space and time may be an avenue of interest for differential diagnosis, such as balance assessment. Maintaining postural control (i.e., balance) requires coordination from multiple body systems, including the vestibular, cognitive, visual, somatosensory, and motor systems (Mancini and Horak, 2010); balance impairments may therefore arise from changes to the aforementioned systems, such as neuropathology and cognitive decline. Greater sway and larger sway velocities have been reported in both mild cognitive impairment and dementia (Bahureksa et al., 2017), suggesting that balance impairments may be a marker of cognitive disorders. This is supported by the reported associations between balance impairments with slower information processing and greater executive dysfunction in Parkinson’s disease (PD) (Fernandes et al., 2016).

With the advent of accelerometer-based wearable technology, conducting balance assessments in constrained settings such as a clinic is increasingly feasible (Mancini et al., 2011b, 2012b). Accelerometer-based balance characteristics are reported as useful measures of postural instability in neurodegenerative populations such as PD (Mancini et al., 2011a). Balance impairments may therefore be useful markers of neurodegenerative disease type and progression, with measures of sway jerkiness (i.e., jerk) in the mediolateral direction significantly impaired in PD compared to controls (Mancini et al., 2012a), and jerk, root mean square (RMS; the magnitude of accelerometer traces), and ellipsis (the area which includes 95% of the mediolateral and anteroposterior accelerometer trajectories) increasing as the disease progresses (Mancini et al., 2012a; Pantall et al., 2018). However, there is a dearth of research examining the ability of balance assessment to discriminate between dementia disease subtypes, with only clinical measures of balance assessment used to report worse balance performance in LBD compared to AD and in PDD compared to DLB (Allan et al., 2005; Fritz et al., 2016; Scharre et al., 2016).

As such, the primary aims of this study were to (1) examine differences in the accelerometer-derived balance characteristics between dementia disease subtypes (i.e., AD, DLB, and PDD) and (2) between dementia disease subtypes and normal aging. A secondary aim was to (3) explore the associations between clinical and cognitive characteristics with balance characteristics in dementia disease subtypes. We hypothesize that (1) Lewy body disease groups (i.e., DLB and PDD) will demonstrate significantly larger jerk, RMS, and ellipsis compared to AD; (2) all dementia disease subtypes will have significantly worse postural instability compared to controls; and (3) slower information processing, greater executive dysfunction, worse motor performance, and lower balance confidence will be significantly correlated with impaired balance characteristics in all dementia disease subtypes.

## Materials and Methods

### Participants

Participants with probable mild cognitive impairment (MCI) or probable dementia due to AD, DLB, and PDD and older adult controls were recruited to the GaitDem Study at Newcastle University. Participants were identified by clinicians in old age psychiatry, geriatric medicine, or neurology services, recruited from a local research case register (the North East DeNDRoN Case Register), or *via* ongoing research studies. The inclusion/exclusion criteria can be found elsewhere (Mc Ardle et al., 2019). All participants had capacity to consent and provided written informed consent. The NHS Local Research Ethics Committee, Newcastle and North Tyneside 1, approved this study.

The disease diagnosis of all participants was verified by two independent clinicians *via* review of medical notes and assessments; disagreements were adjudicated by a third clinician. The relevant diagnostic criteria for dementia due to AD (McKhann et al., 2011), DLB (McKeith et al., 2017), and PDD (Emre et al., 2007) and for MCI due to AD (Albert et al., 2011), DLB (McKeith et al., 2020), and PDD (Litvan et al., 2012) were applied.

### Clinical and Cognitive Assessment

Sex, age, height, and body mass were recorded. Dementia disease stage was assessed with the Clinical Dementia Rating Scale (CDR) (Morris, 1997). Premorbid IQ was measured with the National Adult Reading Test (NART) (Nelson and Willison, 1991). Comorbidities were assessed with the Cumulative Illness Rating Scale – Geriatrics (CIRS-G) (Linn et al., 1968), while motor disease severity was determined using the Movement Disorders Society Unified Parkinson’s Disease Rating Scale (MDS-UPDRS) (Goetz et al., 2008). Functional dependence was assessed using the Bristol Activities of Daily Living Scale (BADLS) (Bucks et al., 1996). Balance confidence was measured using the Activities Balance Confidence (ABC) Scale (Powell and Myers, 1995). Faller status was recorded (i.e., if the participant had experienced a fall within the previous 12 months).

Global cognition was measured using both the standardized Mini Mental State Examination (sMMSE) (Molloy and Standish, 1997) and the Addenbrooke’s Cognitive Examination III (ACE-III) (Noone, 2015), which has subscales measuring attention, language, memory, fluency, and visuospatial abilities. Information processing speed was assessed using the Trail Making Test A (TMT-A) (Bowie and Harvey, 2006). The F-A-S Verbal Fluency test assessed verbal fluency and executive function (Borkowski et al., 1967), and the computerized simple reaction time test measured attention.

### Balance Assessment

A small accelerometer-based wearable (Axivity AX3, York, United Kingdom; dimensions, 23.0 mm × 32.5 mm × 7.6 mm; weight, 11 g; accuracy, 20 ppm; sampling frequency, 100 Hz) was attached to the participants’ lower back in the L5 position using a double-sided hydrogel adhesive and a Hypafix medical plaster. Participants were asked to stand with heels 10 cm apart, maintaining an upright position with arms by their sides and eyes open for 2 min. Participants wore shoes during the assessment. Researchers stood close by in case of adverse events.

Following assessment, the data were downloaded to a computer and processed with a customized MATLAB^®^ script. Accelerations in the anteroposterior and mediolateral planes were of particular interest. Data were filtered using fourth-order zero phase, low-pass Butterworth filter. The cutoff frequency was 3.5 Hz (Del Din et al., 2015). Data were transformed to a horizontal–vertical coordinate system, following which the balance outcomes were extracted in the mediolateral, anteroposterior, and combined directions.

### Balance Characteristics

Seven balance characteristics were derived. Three characteristics related to jerk in the mediolateral, anteroposterior, and combined directions (i.e., the rate of change of acceleration, considered a measure of dynamic stability) (Mancini et al., 2011b). Three characteristics corresponded to RMS in the mediolateral, anteroposterior, and combined directions (i.e., the magnitude of accelerometer traces) (Mancini et al., 2011b). Ellipsis was also derived (i.e., the area which includes 95% of the mediolateral and anteroposterior acceleration trajectories) (Del Din et al., 2015). Data were normalized over the duration of the standing balance test to account for any differences in standing time.

### Data Analysis

Normality of data was assessed using the Shapiro–Wilk test and inspection of the histograms and box plots. Chi-squared tests identified differences between groups for sex and faller status. Kruskal–Wallis tests and one-way analysis of variance (ANOVA) examined differences between groups for all demographic, cognitive, and clinical variables. Mann–Whitney *U* tests and independent *t* tests identified where the differences lay between groups. As all balance characteristics were not normally distributed, Kruskal–Wallis tests and Mann–Whitney *U* tests were used to identify differences between groups. Bonferroni corrections (*p* ≤ 0.007) were applied to account for multiple comparisons. There was one significant outlier in the control group; we assessed group differences with and without the outlier and found no difference to our interpretation of results, so we retained this participant. Receiver operating characteristics and area under the curve (AUC) were used to determine the accuracy of discrete balance characteristics and were interpreted as follows: 0.5–0.7 = low accuracy, 0.7–0.9 = acceptable accuracy, and 0.9–1 = high accuracy. As the data were not normally distributed, Spearman’s correlations were used to explore associations between balance impairments and the demographic, clinical, and cognitive measures.

## Results

### Demographics

One hundred twenty-five participants were recruited to the study; 97 participants were included in this analysis. The reasons for exclusion were as follows: clinical diagnosis other than AD, DLB, PDD, or control (vascular dementia = 7, non-dementia = 4, control with suspected cognitive impairment = 1), withdrawal from the study (*n* = 3), and inability to complete the balance assessment (*n* = 13).

As the dementia disease groups included people with MCI or dementia (see Table 1), we initially examined the differences in balance characteristics within each dementia disease group, comparing MCI and dementia. As there were no significant differences found between MCI and dementia within each subtype, it was deemed feasible to include both disease stages within each dementia disease group (i.e., AD, DLB, and PDD). The demographics and clinical and cognitive information are illustrated in Table 1, with significant between-group differences reported.

**TABLE 1:** Demographics and clinical and cognitive data for dementia disease groups and controls.

	Controls	AD	DLB	PDD	*p*	Between-group differences
*n*	27	31	26	13		
Age (years)	74 ± 9	77 ± 6	76 ± 6	79 ± 6	0.326	
Sex (male/female)	11/16	14/17	22/4	12/1	**<0.001**	a, b, c, d, e
NART	123 (117–126)	117 (101–125)	116 (101–124)	121 (105–124)	**<0.001**	a, b, c
CIRS-G	4 (0–11)	8 (3–16)	10 (5–16)	10 (3–17)	**<0.001**	a, b, c, d, e
MDS-UPDRS III	1 (0–11)	7 (0–19)	26 (0–57)	40 (20–70)	**<0.001**	a, b, c, d, e
Faller status (%)	20%	45%	59%	69%	**0.009**	a, b, c
ABC (%)	94 (52–100)	90 (37–100)	86 (42–100)	75 (21–94)	**<0.001**	a, b, c, e
% Mild cognitive impairment	N/a	39	33	46	0.732	
% Dementia	N/a	61	67	54	0.732	
sMMSE (/30)	30 (25–30)	23 (14–29)	24 (16–30)	24 (12–30)	**<0.001**	a, b, c
ACE-III Total (/100)	97 (87–100)	74 (48–90)	75 (15–95)	79 (49–95)	**<0.001**	a, b, c
ACE-III Attention (/18)	18 (17–18)	14 (6–18)	15 (8–18)	14 (7–18)	**<0.001**	a, b, c
ACE-III Memory (/26)	25 (19–26)	14 (6–23)	17 (0–26)	20 (9–26)	**<0.001**	a, b, c, d, e
ACE-III Fluency (/14)	13 (5–14)	9 (2–13)	8 (3–13)	7 (2–12)	**<0.001**	a, b, c
ACE-III Language (/26)	26 (24–26)	23 (11–26)	23 (0–26)	25 (17–26)	**<0.001**	a, b, c
ACE-III Visuospatial (/16)	16 (13–16)	14 (6–16)	12 (0–16)	11 (9–16)	**<0.001**	a, b, c, d, e
FAS (*n*)	47 (29–75)	35 (3–61)	31 (7–58)	19 (11–48)	**<0.001**	a, b, c, e
TMT-A (s)	30 (19–65)	51 (29–306)	109 (28–835)	95 (24–955)	**<0.001**	a, b, c, d, e
RT Single Task (ms)	373 (291–493)	415 (287–773)	446 (287–1,071)	558 (387–3,792)	**<0.001**	a, b, c, d, e

*Data displayed as mean ± SD were analyzed using one-way ANOVA and *post hoc t* tests. Data displayed as median (min–max) were analyzed using Kruskal–Wallis tests and *post hoc* Mann–Whitney *U* tests. Significant differences are as follows: a = controls vs. AD, b = controls vs. DLB, c = controls vs. PDD, d = AD vs. DLB, and e = AD vs. PDD. AD, Alzheimer’s disease; DLB, dementia with Lewy bodies; PDD, Parkinson’s disease dementia; NART, National Adult Reading Test; CIRS-G, Cumulative Illness Rating Scale – Geriatrics; MDS-UPDRS III, Movement Disorders Society Unified Parkinson’s Disease Rating Scale; ABC, Activities Balance Confidence Scale; sMMSE, standardized Mini Mental State Examination; ACE-III, Addenbrooke’s Cognitive Examination III; FAS, FAS Verbal Fluency Test; TMT-A, Trail Making Task Part A; RT, reaction time. Bold p-values indicate statistically significant results.*

### Differences in Balance Characteristics Between Dementia Disease Subtypes

**FIGURE 1:**
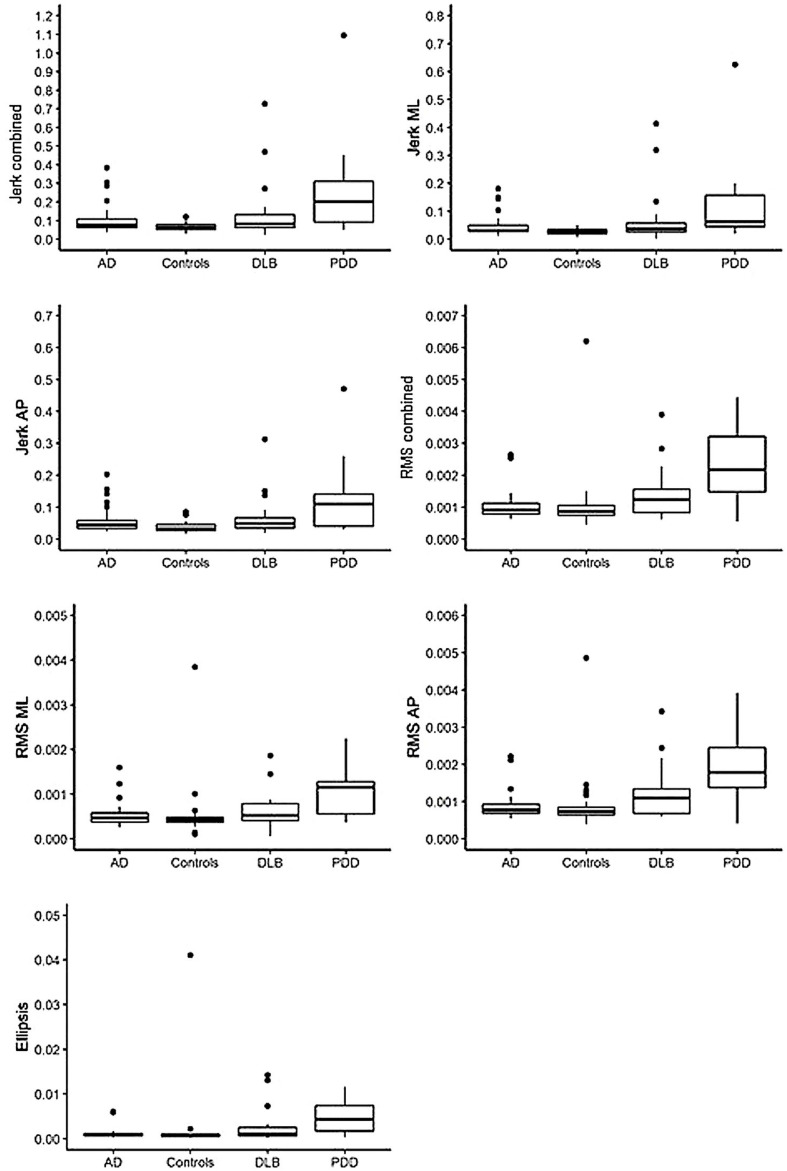
Illustration of balance data across dementia disease subtypes and controls. AD, Alzheimer’s disease; DLB, dementia with Lewy bodies; PDD, Parkinson’s disease dementia; ML, mediolateral; AP, anteroposterior; RMS, root mean square. Dots denote outliers.

Compared to the AD group, the PDD group demonstrated significantly larger jerk in the combined, anteroposterior (AP), and mediolateral (ML) directions (see Tables 2, 3 for statistical significance), larger RMS in the combined, ML, and AP directions, and larger ellipsis (see Figure 1). They also demonstrated significantly larger RMS ML compared to the DLB group. No differences were found between the AD and DLB groups. When Bonferroni corrections were applied, only differences in RMS, RMS AP, and ellipsis remained statistically significant between the AD and PDD groups. ROC curve analysis demonstrated acceptable–excellent accuracy to discriminate PDD from AD (AUC = 0.71–0.82), acceptable accuracy to discriminate PDD from DLB (AUC = 0.69–0.74), and low accuracy to discriminate DLB from AD (AUC = 0.50–0.65) for all balance characteristics (see Table 3).

**TABLE 2:** Balance differences between dementia disease groups and controls.

	Controls	Alzheimer’s disease	Dementia with Lewy bodies	Parkinson’s disease dementia	*p*	Between-group differences
*n*	27	31	26	13		
Jerk combined (m^2^ s^–6^)	0.063 (0.027–3.942)	0.075 (0.038–0.383)	0.080 (0.023–0.726)	0.203 (0.050–1.10)	**0.003**	a
Jerk ML (m^2^ s^–6^)	0.029 (0.006–2.669)	0.030 (0.013–0.180)	0.037 (0.002–0.414)	0.062 (0.019–0.625)	0.008	
Jerk AP (m^2^ s^–6^)	0.035 (0.016–1.274)	0.044 (0.024–0.202)	0.047 (0.019–0.312)	0.109 (0.031–0.471)	**0.002**	a
RMS combined (m s^–3^)	0.0008 (0.0004–0.0061)	0.0009 (0.0006–0.0027)	0.0012 (0.0006–0.0039)	0.0022 (0.0005–0.0044)	**0.001**	a, b
RMS ML (m s^–3^)	0.0004 (0.0001–0.0039)	0.0005 (0.0003–0.0016)	0.0005 (0.0001–0.0019)	0.0012 (0.0004–0.0023)	**0.006**	a, b
RMS AP (m s^–3^)	0.0007 (0.0004–0.0049)	0.0008 (0.0006–0.0022)	0.0011 (0.0006–0.0034)	0.0018 (0.0004–0.0039)	**0.001**	a, b
Ellipsis (m^2^ s^–5^)	0.0007 (0.0001–0.0410)	0.0008 (0.0004–0.0061)	0.0011 (0.0001–0.0142)	0.0043 (0.0003–0.0116)	**0.001**	a, b

*Data are reported as median (minimum–maximum). Kruskal–Wallis tests were used to identify differences between groups. Mann–Whitney *U* tests were used to identify where differences lay. Bonferroni correction (*p* < 0.007) applied. Significant differences are as follows: a = PDD vs. controls and b = PDD vs. AD. ML, mediolateral; AP, anteroposterior; RMS, root mean square. Bold p-values indicate statistically significant results.*

**TABLE 3:** Area under the curve values of discrete balance characteristics between groups based on receiver operator curve analysis.

	AD vs. DLB				AD vs. PDD				PDD vs. DLB			
	Area	*p*	CI lower	CI upper	Area	*p*	CI lower	CI upper	Area	*p*	CI lower	CI upper
Jerk combined	0.531	0.689	0.377	0.685	0.73	0.017	0.55	0.909	0.698	0.046	0.511	0.886
Jerk ML	0.545	0.564	0.391	0.699	0.722	0.021	0.538	0.907	0.695	0.049	0.512	0.878
Jerk AP	0.535	0.654	0.381	0.689	0.715	0.026	0.531	0.898	0.692	0.053	0.502	0.883
RMS combined	0.655	0.045	0.503	0.807	0.811	**0.001**	0.63	0.992	0.734	0.019	0.546	0.922
RMS ML	0.574	0.336	0.418	0.731	0.779	**0.004**	0.608	0.95	0.731	0.02	0.546	0.915
RMS AP	0.656	0.044	0.505	0.807	0.824	**0.001**	0.649	0.999	0.743	0.015	0.557	0.928
Ellipsis	0.624	0.109	0.471	0.778	0.804	**0.002**	0.624	0.984	0.71	0.034	0.524	0.896

	**AD vs. Controls**				**DLB vs. Controls**				**PDD vs. Controls**			
	**Area**	***p***	**CI lower**	**CI upper**	**Area**	***p***	**CI lower**	**CI upper**	**Area**	***p***	**CI lower**	**CI upper**

Jerk combined	0.652	0.047	0.51	0.794	0.682	0.023	0.534	0.831	0.821	**0.001**	0.667	0.974
Jerk ML	0.626	0.1	0.481	0.771	0.67	0.034	0.518	0.821	0.792	**0.003**	0.613	0.971
Jerk AP	0.675	0.022	0.534	0.816	0.687	0.02	0.539	0.834	0.838	**0.001**	0.707	0.968
RMS combined	0.558	0.45	0.406	0.71	0.695	0.015	0.552	0.839	0.818	**0.001**	0.646	0.989
RMS ML	0.584	0.272	0.435	0.734	0.628	0.109	0.472	0.785	0.795	**0.003**	0.626	0.963
RMS AP	0.56	0.431	0.408	0.713	0.684	0.022	0.538	0.83	0.823	**0.001**	0.657	0.99
Ellipsis	0.578	0.307	0.43	0.727	0.668	0.036	0.517	0.819	0.826	**0.001**	0.664	0.988

*Bonferroni correction (*p* < 0.007) applied. Bold p-values indicate statistically significant results.*

### Differences in Balance Characteristics Between Dementia Disease Subtypes and Controls

Compared to the controls, both the PDD and DLB groups demonstrated significantly larger jerk in the combined, AP, and ML directions, larger RMS in the combined and AP directions, and larger ellipsis (see Tables 2, 3 and Figure 1). The PDD group also demonstrated greater RMS ML compared to the controls. The AD group had greater jerk AP compared to the controls. When Bonferroni corrections were applied, only differences between the controls and PDD for all characteristics remained statistically significant. ROC curve analysis demonstrated excellent accuracy to discriminate PDD (AUC = 0.79–0.83) from the controls and low accuracy to discriminate AD (AUC = 0.55–0.67) and DLB (AUC = 0.62–0.68) from the controls for all balance characteristics (see Table 3).

### Associations Between Balance Characteristics and Clinical and Cognitive Measures in Dementia Disease Subtypes

#### Alzheimer’s Disease

**FIGURE 2:**
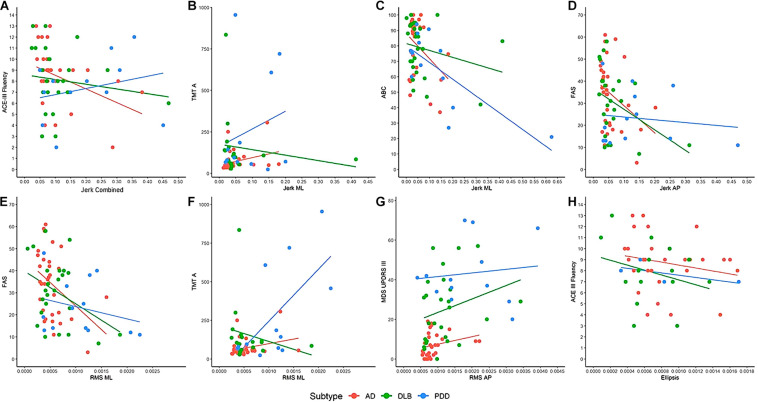
Examples of the associations between clinical and cognitive variables with balance characteristics. **(A)** ACE-III Fluency associated with jerk vertical in AD (rho = 0.422, *p* = 0.018). **(B)** TMT-A associated with jerk ML in AD (rho = 0.416, *p* = 0.020) and DLB (rho = 0.539, *p* = 0.035). **(C)** ABC associated with jerk ML in PDD (rho = 0.578, *p* = 0.039). **(D)** FAS associated with jerk AP in AD (rho = 0.377, *p* = 0.037) and DLB (rho = 0.433, *p* = 0.035). **(E)** FAS associated with RMS ML in AD (rho = 0.374, *p* = 0.038). **(F)** TMT-A is associated with RMS ML in PDD (rho = 0.566, *p* = 0.044). **(G)** MDS UPDRS-III is associated with RMS AP in AD (rho = 0.428, *p* = 0.018). **(H)** ACE-III Fluency is associated with ellipsis in AD (rho = 0.378, *p* = 0.036). AD, Alzheimer’s disease; DLB, dementia with Lewy bodies; PDD, Parkinson’s disease dementia; ML, mediolateral; AP, anteroposterior; RMS, root mean square; ACE-III, Addenbrooke’s Cognitive Examination; TMT-A, Trail Making Test A; ABC, Activities Balance Confidence Scale; FAS, FAS Verbal Fluency Test; MDS-UPDRS III, Movement Disorders Society Unified Parkinson’s Disease Rating Scale. Dots denote outliers.

In AD, older age was associated with greater combined (rho = 0.424, *p* = 0.018) and AP RMS (rho = 0.438, *p* = 0.014) and larger ellipsis (rho = 0.404, *p* = 0.024). Greater motor problems, as measured by UPDRS-III, was associated with greater RMS AP (rho = 0.428, *p* = 0.018; see Figure 2). Worse verbal fluency, as measured by ACE-III Fluency, was significantly associated with greater combined (rho = 0.422, *p* = 0.018), ML (rho = 0.406, *p* = 0.024), and AP jerk (rho = 0.426, *p* = 0.017), greater combined (rho = 0.373, *p* = 0.039) and ML RMS (rho = 0.369, *p* = 0.041), and larger ellipsis (rho = 0.378, *p* = 0.036). Similar findings were found between the FAS verbal fluency test with jerk AP (rho = 0.377, *p* = 0.037) and RMS ML (rho = 0.374, *p* = 0.038). Slower information processing, as measured by TMT-A, was significantly associated with greater combined (rho = 0.461, *p* = 0.009), ML (rho = 0.416, *p* = 0.020), and AP jerk (rho = 0.454, *p* = 0.010).

#### Dementia With Lewy Bodies

In DLB, better visuospatial abilities (rho = 0.423, *p* = 0.035), as measured by the ACE-III visuospatial subscale, and quicker information processing (rho = 0.539, *p* = 0.026) were associated with greater jerk ML (see Figure 2). Worse verbal fluency was associated with greater jerk AP (rho = 0.433, *p* = 0.035).

#### Parkinson’s Disease Dementia

In PDD, worse balance confidence, as measured by the ABC scale, was associated with greater jerk ML (rho = 0.578, *p* = 0.039; see Figure 2). Slower information processing was associated with greater RMS ML (rho = 0.566, *p* = 0.044).

## Discussion

This is the first study to examine the differences in accelerometer-derived balance characteristics between dementia disease subtypes and normal aging. The key results demonstrate that people with PDD could be discriminated with acceptable accuracy from both people with AD and cognitively intact older adults based on balance impairments. However, differentiating between DLB and AD is more clinically challenging, and therefore, discriminative markers for these groups are considered a research priority (Kane et al., 2018). Our results demonstrated that balance assessment could not acceptably discriminate DLB from any other subtype, nor could it differentiate normal aging from AD or DLB.

### Balance Assessment as a Differential Marker of Dementia Disease Subtype

In partial agreement with hypothesis 1, the PDD group demonstrated greater RMS in the combined and mediolateral directions and larger ellipsis compared to people with AD. This is consistent with findings from clinical measures (Allan et al., 2005; Fritz et al., 2016; Scharre et al., 2016). However, no differences were found between DLB and either AD or PDD once multiple comparison corrections were applied. Although this is the first study to quantitatively examine balance impairments across dementia disease subtypes, these findings contrast with findings of significant differences in balance performance between all three dementia subtypes when assessed with an observational clinical measure (i.e., the Tinetti Balance subscale) (Allan et al., 2005; Fritz et al., 2016; Scharre et al., 2016). Confirmation bias introduced by clinical measures may explain the discrepancy in the findings, as examiners may subjectively expect greater balance problems in cohorts with clinically defined motor problems such as LBD (Emre et al., 2007; McKeith et al., 2017) compared to conditions that are not traditionally considered to have motor impairments such as AD (Allan et al., 2005). Based on our results, we do not recommend an eyes-open accelerometer-based balance assessment as a differential tool for AD and DLB.

### Balance Assessment as a Differential Marker of Cognitive Impairment

In disagreement with hypothesis 2, only the PDD group demonstrated significant differences across all balance characteristics compared to cognitively intact controls. These results support previous findings that accelerometer-based balance assessment is useful for differentiating PD from normal aging and for monitoring disease progression and cognitive decline in people with PD (Mancini et al., 2012a; Schoneburg et al., 2013; Del Din et al., 2015; Pantall et al., 2018). However, static eyes-open balance assessment did not appear significantly impaired in other dementia disease subtypes and, therefore, may not be a good marker of general cognitive impairment; this contrasts previous literature (Bahureksa et al., 2017). It should be noted that our cohort was a predominately mild dementia group, composed of both MCI and dementia participants, and this may have impacted our results. The review of Bahureksa et al. (2017) found limited differences in static balance performance in the MCI groups compared to normal aging in eyes-open conditions, suggesting that these groups use visual feedback to appropriately maintain their postural stability. As such, balance differences under these conditions may be apparent in the later stages of dementia disease. However, this is not useful to support early diagnosis of cognitive impairment, which is required to better manage the condition, ensure patients and carers can appropriately plan for the future, and to improve researchers’ understanding of early disease stages in order to develop novel targets for therapeutics (Kenigsberg et al., 2016). We therefore suggest alternative motor performance measures to support differential diagnosis. For example, gait assessment has demonstrated acceptable accuracy to discriminate AD and DLB and may therefore be an effective easy-to-use supportive diagnostic marker (Mc Ardle et al., 2019, 2020).

### Relationships Between Discrete Clinical and Cognitive Characteristics With Balance

To aid interpretation of the results, we examined associations between clinical and cognitive measures with balance performance. Partially agreeing with hypothesis 3, correlations were found between cognitive impairments and balance impairments. This was most apparent in the AD group, with slower information processing and worse verbal fluency (often considered a measure of executive function) (Williams-Gray et al., 2009) associated with poor balance performance. This is supported by the literature in PD (Fernandes et al., 2016), suggesting that balance relies on these discrete cognitive processes to maintain postural stability. For example, executive function may be important for planning and set-shifting during standing balance and may inhibit inappropriate postural responses (Schoneburg et al., 2013). Interestingly, associations between greater motor disease and worse balance were only found in the AD group, and associations between worse balance confidence and balance were only found in the PDD group; however, trends indicated similar directionality in all groups (see Figure 2). As the PDD group demonstrated significantly slower information processing, worse verbal fluency, greater motor disease burden, and worse balance confidence compared to the AD group, this may somewhat account for their poorer balance performance. Overall, static balance may not be challenging to cognitive and motor abilities, particularly in lab-based environments which lack complexities experienced in the real word that may increase cognitive demands, such as constrained spaces, moving objects, and visual stimuli. The results may have been different if static balance performance was examined under different conditions. For example, eyes-closed static balance assessments increase reliance on the vestibular system and decreases compensatory visual and cognitive input for the maintenance of balance, potentially revealing greater balance impairments (Schoneburg et al., 2013; Bahureksa et al., 2017). Similarly, static balance assessments on uneven surfaces have demonstrated significantly worse balance in AD compared to normal aging (Suttanon et al., 2012). Standing statically on uneven surfaces, such as foam, requires consistent and quick postural adaptions to maintain balance (yeun Lee et al., 2011) and may reveal significant impairments when participants have discrete cognitive deficits, such as slower information processing that slows their anticipatory postural adjustments. Other studies have also employed cognitive dual tasks to static balance assessment in people with AD (Manckoundia et al., 2006). This places competition on cognitive resources, as participants are trying to maintain balance while carrying out an additional cognitive task, and produces greater balance impairments compared to single-task balance assessments. Future research could consider the impact of different conditions such as those outlined on balance in people with cognitive impairments. However, the findings from this study suggest that eyes-open static balance assessments will not be useful additions to the diagnostic toolkit.

### Limitations and Directions for Future Research

A main strength of this study was that all participants’ diagnoses were confirmed by clinicians’ consensus based on clinical notes and well-characterized clinical and cognitive profiles. However, while this lends confidence to our results, diagnosis of dementia subtype can only be confirmed postmortem, which was beyond the scope of this study. We also looked at groups across the spectrum of cognitive impairment, which was deemed feasible as the MCI and dementia participants were indistinguishable in terms of balance impairments. However, there are limits to this approach; although we applied validated criteria for MCI due to dementia disease subtype (Litvan et al., 2012; McKeith et al., 2020), not all MCI participants may progress to dementia, and it was beyond the scope of this study to determine whether participants with MCI due to Lewy body disease went on to develop DLB or PDD. Additionally, although this is the first study of its kind, our sample size was small, causing difficulties to the generalizability of the findings, and outliers may have affected the results. Raw data were checked to ensure that outlier data were correct, highlighting the skewed distribution of balance performance. As our data were not normally distributed, we used non-parametric analysis to explore differences between groups. This limited our ability to account for potential confounders, such as age and sex, which may have improved our interpretation of results. Larger studies are required to account for these issues with generalizability and skewed distribution and would strengthen the findings described here. Our results suggest that a 2-min static eyes-open balance assessment is not a useful differential marker of dementia disease subtype or cognitive impairment. Studies in PD have indicated that balance impairments may be time-dependent, with shorter bouts producing more sensitive results (Del Din et al., 2015). However, we examined postural stability across different bout lengths (e.g., <30 and <60 s) and found that it did not change our interpretation. Additionally, we did not assess visual acuity in this study. As vision plays a significant role in the maintenance of balance, our lack of insight into participants’ visual acuities is considered a limitation (Hill et al., 2016; Baydan et al., 2020; Hunter et al., 2020). To ensure participant safety, a researcher stood close to the participants who were visibly unstable or worried about their balance; this may also have influenced the results as it provided more security and confidence for the participants. It should be noted that static balance is only one element of balance; dynamic balance assessments require faster postural adjustments and may be significantly more compromised by cognitive impairments (Liaw et al., 2009). As we did not assess dynamic balance, we cannot draw conclusions on the efficacy of such assessments to detect cognitive impairment or dementia disease subtype. Finally, there is growing interest in classification methods, such as machine learning, which involve combining different disease features and assessing the best combinations for discriminating diseases. In the future, these methods could be applied in larger studies to combinations of balance characteristics, such as those described in this article, or to a combination of balance characteristics with other motor performance measures, such as gait or dynamic balance performance.

## Conclusion

In conclusion, this study found that static eyes-open balance assessments could only acceptably differentiate PDD from AD and controls. Static eyes-open balance assessment is not a useful differential marker of AD and DLB or for distinguishing general cognitive impairment from normal ageing. In line with previous work in PD, associations were found between slower information processing and greater executive dysfunction with balance impairments, suggesting that cognition may play a role in safely maintaining balance. Future research could examine the impact of alternative conditions, such as eyes closed or dual tasks, on balance across dementia disease subtypes as this may prove a more fruitful endeavor.

## Data Availability Statement

The datasets generated and analyzed for this study can be made available by request with permission of the GaitDem Data Management team (lynn.rochester@ncl.ac.uk).

## Ethics Statement

The studies involving human participants were reviewed and approved by the NHS Local Research Ethics Committee, Newcastle and North Tyneside 1. The patients/participants provided their written informed consent to participate in this study.

## Author Contributions

LR, BG, AT, LA, and RM contributed to the conception and design of this study. RM and LA collected the data for this study. RM, CB, SD, and LA organized the database. RM and SP performed the statistical analysis and wrote the first draft of the manuscript. All authors contributed to the manuscript revision and read and approved the submitted version.

## Conflict of Interest

The authors declare that the research was conducted in the absence of any commercial or financial relationships that could be construed as a potential conflict of interest.
